# Nanobodies: Prospects of Expanding the Gamut of Neutralizing Antibodies Against the Novel Coronavirus, SARS-CoV-2

**DOI:** 10.3389/fimmu.2020.01531

**Published:** 2020-06-23

**Authors:** Rocktotpal Konwarh

**Affiliations:** ^1^Department of Biotechnology, Addis Ababa Science and Technology University, Addis Ababa, Ethiopia; ^2^Centre of Excellence-Nanotechnology, Addis Ababa Science and Technology University, Addis Ababa, Ethiopia

**Keywords:** COVID-19, SARS-CoV-2, neutralizing antibody, nanobodies, spike protein

With more than 6.9 M confirmed cases and ~400 K deaths as on June 8, 2020 (1), COVID-19, ushered in by the SARS-CoV-2 has projected itself as a microscopic-holocaust, much more sinister than those portrayed in the SciFi movies. Asymptomatic transmission of the virus has been projected as the Achilles' heel in the context of the current control strategies of the pandemic (2, 3). Reports on undiagnosed deep vein thrombosis among patients, succumbing to the viral assault (4) and demonstration of direct infection of human blood vessel and kidney organoids (5) have triggered huge hue and cry. The extreme high transmissibility of the virus, bracketed together with current absence of population immunity and occurrence of stark clinical consequences projects the swift advancement in effective therapeutic stratagems as the need of the hour. Needless to say, researchers, across the globe, are beavering to devise appropriate diagnostic and therapeutic strategies. The various nucleic acid based detection-approaches like PCR, isothermal nucleic acid amplification-based methods, CRISPR/Cas platforms as well as immunoassay based point-of-care lateral flow tests are marked with respective pros and cons (6, 7). On the other hand, strategies of inhibiting the viral fusion/entry, disrupting the replication pathway, suppressing the inflammatory response, using convalescent plasma treatment and vaccine development have been at the forefront of recent research (8). The success lies in our comprehensive understanding of the “*biochemically and genetically guileful”* virus. At this juncture, it is relevant to mention that long-term development of appropriate antibody and other protein therapeutics to effectively bind and neutralize the viral infection is imperative. This would be significant in case the researchers need to buy excess time to ensure befitting vaccine discovery and development. Such therapeutics could possibly provide an alternative/additional way to assist those people who might show unresponsiveness to vaccines (as, exemplified by many in the elderly population) or do not obtain vaccine. Amidst the current hay-wired situation, the recent communiqué from Israeli Defense Minister Natfali Bennet about the successful isolation of a “*monoclonal neutralizing antibody*” with potency to “*neutralize [disease] inside carriers” bodies*' by the scientists in the Israel Institute for Biological Research has ushered in new waves of hope (9).

Prior to getting ahead, it would be prudent to recapitulate the general aspects of the lifecycle of the highly pathogenic human coronaviruses (CoVs) (10) (Figure 1A). Talking about the viral pathogenesis, the receptor binding domains (RBD) of the spike (S) glycoprotein interact with the human angiotensin-converting enzyme 2 (ACE2)- the receptor that *invites* SARS-CoV and SARS-CoV-2 into human cells (Figure 1Ba). The presence of a furin cleavage site at interfacial zone of the S_1_/S_2_ subunits of the SARS-CoV-2 S glycoprotein demarcates the virus from SARS-CoV and SARS-related CoVs (13). Precise understanding of the SARS-CoV-2 S ectodomain trimer is envisaged to be instrumental in developing vaccines, therapeutic antibodies and diagnostics. The prospective targets of neutralizing antibodies (nAbs) against human pathogenic CoVs are depicted in Figure 1Bb. Monoclonal antibodies (mAbs), functional antigen-binding fragment (Fab), single-chain variable region fragment (scFv), and single-domain antibodies (nanobodies or Nbs) have been assessed against various human CoVs (14–19). Jiang et al. (10) have recently reviewed the development of SARS-CoV- and MERS-CoV-specific nAbs, while literature reports on nAbs against SARS-CoV-2 are comparatively scanty. Previous studies on neutralization with anti-SARS-CoV-1 RBD and anti-MERS-CoV RBD antibodies had unveiled a premature switching from the pre-fusion to post-fusion conformation following a closure of the receptor binding site and trapping the RBD in “up” conformation (20–22). The structure of CR3022, an antibody derived from a convalescent SARS patient, in complex with the RBD of the S protein at a resolution of 3.1 Å was recently reported (23). Interestingly, a cross-reactive interaction between SARS-CoV-2 and SARS-CoV was evinced by the elucidation that a highly conserved but cryptic, epitope, distal from the receptor binding site is targeted by CR3022. However, at least two RBDs on the trimeric S protein in the “up” conformation and slight rotation are prerequisites to access the binding epitope by CR3022. The authors proposed that albeit, the CR3022 fails to neutralize SARS-CoV-2 *in vitro*, the epitope could plausibly confer *in vivo* protection. On a similar vein, researchers have resorted to the use of SARS-CoV-2 S murine polyclonal antibodies for the inhibition of SARS-CoV-2 S mediated entrance into cells (13). The study vouched that vaccination could elicit cross-neutralizing antibodies, targeting the conserved S epitopes.

**Figure 1:**
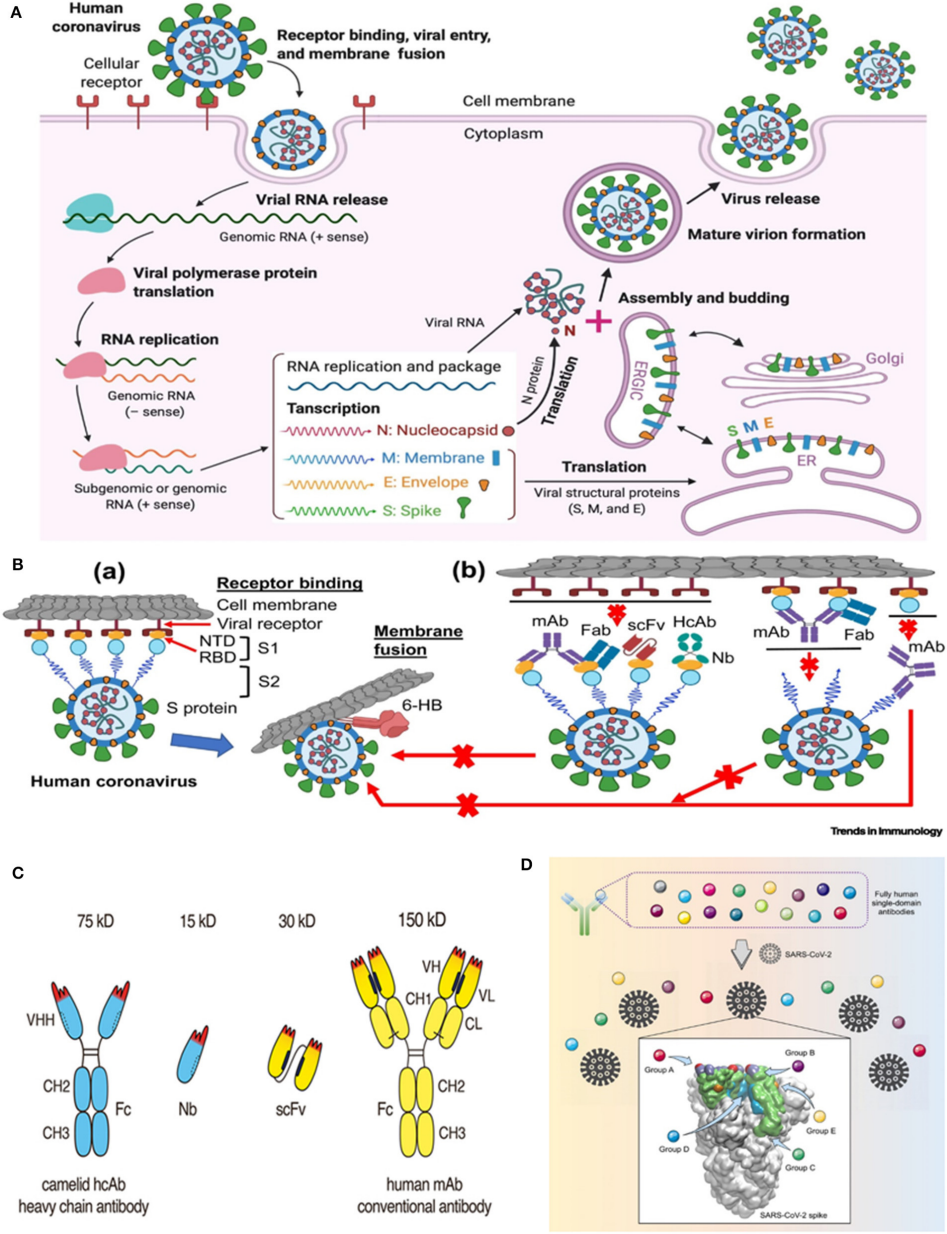
Life cycle of highly pathogenic human coronaviruses (CoVs) and specific neutralizing antibodies (nAbs) against these coronaviruses. **(A)** Life cycle of highly pathogenic human CoVs. These CoVs enter host cells by first binding to their respective cellular receptors [angiotensin-converting enzyme 2 (ACE2) for severe acute respiratory syndrome (SARS)-CoV-2 or SARS-CoV and dipeptidyl peptidase 4 (DPP4) for Middle East respiratory syndrome (MERS)-CoV] on the membranes of host cells expressing ACE2 (e.g., pneumocytes, enterocytes) or DPP4 (e.g., liver or lung cells including Huh-7, MRC-5, and Calu-3) via the surface spike (S) protein, which mediates virus–cell membrane fusion and viral entry. Viral genomic RNA is released and translated into viral polymerase proteins. The negative (–)-sense genomic RNA is synthesized and used as a template to form sub-genomic or genomic positive (+)-sense RNA. Viral RNA and nucleocapsid (N) structural protein are replicated, transcribed, or synthesized in the cytoplasm, whereas other viral structural proteins, including S, membrane (M), and envelope (E), are transcribed then translated in the endoplasmic reticulum (ER) and transported to the Golgi. The viral RNA-N complex and S, M, and E proteins are further assembled in the ER–Golgi intermediate compartment (ERGIC) to form a mature virion, then released from host cells. **(B)** Potential targets of nAbs against SARS-CoV-2 and other pathogenic human CoVs. (a) Human CoV receptor binding and membrane fusion process. The CoV first binds a viral receptor (ACE2 or DPP4) through the receptor-binding domain (RBD) in the S protein, followed by fusion of the virus with cell membranes via the formation of a six-helix bundle (6-HB) fusion core. NTD, N-terminal domain. (b) Potential targets of nAbs on the S protein of human CoVs. Monoclonal antibody (mAb), antigen-binding fragment (Fab), single-chain variable region fragment (scFv), or single-domain antibody [nanobody (Nb) or VHH derived from camelid heavy chain antibody (HcAb)] binds to the RBD, S1 subunit (non-RBD, including NTD), or S2 of the viral S protein, blocking binding between the RBD and the respective receptor (for RBD-targeting nAbs), interfering with the conformational change of S (for S1-targeting nAbs), or hindering S2-mediated membrane fusion (for S2-targeting nAbs), leading to the inhibition of infection with pathogenic human CoVs in the host cells. The figure was created using BioRender (https://biorender.com/). [Reproduced from (10), under the provisions of Creative Commons License, CC BY 4.0, Copyright © 2020 The Author(s). Published by Elsevier Ltd.]. **(C)** Advantageous features of camelid heavy chain antibodies. Heavy chain antibodies are composed of two heavy chains. The target-binding module is composed of a single VHH domain. A recombinant VHH domain, designated nanobody (Nb) is highly soluble and does not show any tendency to associate with other hydrophobic protein surfaces. Conventional antibodies are composed of two heavy and two light chains. The target-binding module is composed of two non-covalently associated variable domains VH and VL. In intact antibodies, the proper orientation of these domains is mediated by a hydrophobic interface and is further stabilized by the disulfide-linked CL and CH1 domains. A pair of VH and VL domains can be linked genetically into a single-chain variable fragment (scFv) in which the proper orientation of domains is mediated alone by the hydrophobic interface between the two V-domains. [Reproduced from (11), under the provisions of Creative Commons Attribution License (CC BY). Copyright © 2017 Bannas, Hambach and Koch-Nolte]. **(D)** Targeting of diverse epitopes within the SARS-CoV-2 spike protein receptor binding domain (RBD) by human single-domain antibodies, potential therapeutic candidates for COVID-19. [Reproduced from (12) Copyright ©2020 Elsevier Inc., based on the reuse-provisions of Elsevier's COVID-19 Resource Centre].

At this juncture, the germaneness of antibody engineering may be comprehended in the context of continual search for high-affinity antibodies, effective against conserved targets as well as novel therapeutics with attributes like better tumor and tissue penetration and efficient launching of immune effector functions (24). Particularly, in the context of antitumor therapeutics, Bannas et al. (11) had raised concerns about the large-size (150 kDa) dictated practical snag of *in vivo* delivery of conventional antibodies to tumor cells. On the other hand, aggregation and/or mispairing of V-domains due to lower stability and solubility of engineered antibodies- a consequence of intrinsic hydrophobic interactions of VH and VL domains (that constitute the antigen binding fragment (Fab) of IgG antibodies) have been another pertinent issue. As plausible solutions, nanobodies (15 kDa) and nanobody based human heavy chain antibodies (75 kDa) (11) have instigated considerable research impetus. Besides conventional antibodies, camelids produce heavy-chain-only antibodies (HCAbs) with a single variable domain as the target recognition module (25, 26). This single variable domain without an effector domain functions as a single-domain antibody, VHH, or nanobody (Nb) (Figure 1C). Although the prospects of using nanobodies as research and diagnostic tools have been critically and comprehensively assessed (27, 28) and a plethora of nanobodies are currently being placed under pre-clinical or clinical assessments for various diseases like brain tumors, inflammation, lung diseases, as well as autoimmune diseases, paralleling the performance of classical antibodies with nanobodies for therapeutic applications could be bit fiddly (29). Nevertheless, studies have attested the advantages of nanobodies in contrast to conventional antibodies with respect to the former's smaller size, amenability for processing into multiple formats, desirable thermal and chemical stability, high solubility, commendable *in vivo* tissue penetration and targeting, lower susceptibility to steric hindrances (that may otherwise obstruct optimal binding) as well as ability to display antigenic affinity and specificity at par with conventional antibodies (11, 30–34). Prospects of genetically linking to Fc-domains, peptide tags, or other nanobodies as well as site-specific chemical fusion with nanoscale materials, radionuclides, photosensitizers, etc. widen the spectrum of their applications. Furthermore, the expedient attributes of nanobodies and human Fc domains may be combined in chimeric nanobody-heavy chain antibodies, half the size of the conventional antibodies, as mentioned before (11).

Post perusal of the afore-stated, harnessing VHHs as therapeutics against various viral infectious agents seems to be an interesting proposition (35). In this respect, use of VHH against dengue virus (36); hepatitis C virus (37); multiple VHH monovalent candidates against poliovirus (38) and norovirus (39); anti-CXCR4 monovalent and bivalent (40) as well as anti-p24 monovalent and bivalent (41) nanobodies against HIV; VHH bivalent/albumin-linked nanobody against rabies virus (42) and anti-VP6 VHH as an effective prophylactic treatment against rotavirus A-associated diarrhea (43) have been documented. Investigations on the application of nanobodies against respiratory pathogens has also gained pace in recent years. Use of H5N1-HA bivalent nanobody against influenza virus (44), as well as the application of multi-domain antibody MD3606 (generated using diverse camelid single-domain antibodies to influenza virus hemagglutinin) to protect mice against influenza A and B infection post intravenous administration or expression using recombinant adeno-associated vector (32), merit special mention. Similarly, two llama-derived single-domain antibodies with human respiratory syncytial virus (RSV)–neutralizing action have been reported to selectively bind to RSV fusion protein (F) in its pre-fusion state with picomolar affinity (45). Delivering a trimeric nanobody, ALX-0171 (that interacted with antigenic site II of RSV F protein at subnanomolar affinity), prophylactically or therapeutically directly to lungs of cotton rats was effective in down-scaling both nasal and lung RSV titers (46). Stalin Raj et al. (47) had resorted to direct cloning and expression of VHHs of HCAbs from the bone marrow of MERS-CoV–infected Arabian camels and identified several MERS-CoV–specific VHHs or nanobodies. With a prolonged half-life in serum, camel/human chimeric HCAbs were efficacious in endowing protection to mice against MERS-CoV challenge. In a similar vein, the efficacy to target MERS-CoV S RBD using novel neutralizing Nb (NbMS10) and its human-Fc-fused version (NbMS10-Fc) has been documented (48). Remarkably, the Nbs were able to cross-neutralize infections caused by diverse MERS-CoV strains isolated from humans and camels. The Fc-tagged Nb was able to confer complete protection of humanized mice from lethal MERS-CoV assault.

A concerted effort of biologist Michael Rout and chemist Brian Chait has been directed toward selecting high affinity and effective neutralizing nanobodies, interacting with the various non-overlapping target-epitopes of SARS-CoV-2 S (49). The researchers envisage to set-up the appropriate nanobodies as increased level multimers to augment affinity and eventually tune them at the molecular level to better their neutralizing potency. Similarly, researchers from *Protein Production UK*, a project hosted by the Rosalind Franklin Institute in association with Diamond Light Source, UK, have made nanobodies (exhibiting high affinity to the S protein of the SARS-CoV-2), available to scientist at the University of Oxford for deeper delving into the structure of the virus (50). On a stimulating note, scientists from the University of Texas (UT) at Austin, the National Institutes of Health and Ghent University in Belgium have documented the isolation of two potently neutralizing VHHs, targeting the SARS-CoV-1 and MERS-CoV RBDs, respectively (34). Wrapp et al. (34) had resorted to sequential immunization of a llama subcutaneously multiple times with SARS-CoV-1 S and MERS-CoV S protein. Two sequential rounds of panning were executed by phage display using either SARS-CoV-1 S or MERS-CoV S proteins to procure VHHs directed against the S proteins. The researchers successfully isolated seven unique MERS-CoV S and five SARS-CoV-1 S specific VHHs post-sequencing of the positive clones, multiple sequence alignment, and phylogenetic analysis. Following expression in *Pichia pastoris* and purification from yeast medium, the interaction of the purified VHHs with the perfusion-stabilized MERS-CoV S and SARS-CoV-1 S was attested by ELISA. Pertinently, the SARS-CoV-1 RBD-directed VHH could cross-react with the SARS-CoV-2 RBD. A fascinating dimension to the work was the neutralization of the SARS-CoV-2 S pseudotyped viruses by the cross reactive VHH, engineered as a bivalent human IgG Fc-fusion. The plausible scaled up production of the VHH-Fc fusion was attested in a commercial-standard CHO cell system. The MERS VHH-55, SARS VHH-72 and VHH-72-Fc, exhibiting desirable biophysical attributes and potent neutralization potency, could be prospective therapeutic candidates. However, appropriate *in vivo* experimentations as part of preclinical studies are prerequisite.

Retrieval of information from the preprint at *BioRxiv* evinces the successful endeavors of Swiss researchers Walter et al. (51) in identifying 63 unique anti-RBD synthetic nanobodies or *sybodies*, interacting in the context of the full-length SARS-CoV-2 spike ectodomain. Assisted by a prompt *in vitro* selection platform (encompassing ribosome and phage display), the task of selecting the sybodies was accomplished within 12 days. Six of the selected sybodies displayed double-digit nanomolar binding affinity with the viral spike while five of them could inhibit RBD interaction with ACE2. Furthermore, the researchers identified a pair of anti-RBD sybodies that could concomitantly interact with the RBD. It would be interesting to peruse the outcomes of the authors' previously reported NestLink technology (52) based delving of the selection pools to unearth unique sybodies with little off-rates and capacity to identify rare epitopes. The authors are upbeat about plausible therapeutic exploitation of the sybodies for the development of an inhalable drug as useful prophylaxis against COVID-19.

To speak about yet another development, Beroni Group (an international biopharmaceutical enterprise) in concert with Tianjin University in China has recently identified 24 types of nanobodies (post-screening a library with one billion-plus nanobody sequences) for prompt detection and treatment of SARS-CoV-2 (53). Eight of them are directed against the S protein while sixteen of them target the nucleocapsid (N) protein- the latter could find application as a marker in diagnostic assays. Based on approaches of structural biology, computational biology, and protein engineering, the researchers are gearing up to optimize the properties of the nanobodies besides endeavoring to reduce their immunogenicity and augment the therapeutic efficiency by humanizing them. By the same token, researchers from Fudan University and Biomissile Corporation, China have directed their endeavors toward the development of a phage-displayed single-domain antibody library based on embedding naive complementarity-determining regions (CDRs) into framework sites of a human germline immunoglobulin heavy chain variable region (IGHV) allele (12). Their study, encompassing the library-biopanning against SARS-CoV-2 RBD and S1 subunit led to the revelation of fully human single-domain antibodies, displaying low-nanomolar/subnanomolar range affinities toward five distinct epitopes on SARS-CoV-2 RBD (Figure 1D). Amongst the groups of A, B, C, D, and E neutralizing antibodies, the group D members, n3088 and n3130 could target a “cryptic” epitope, positioned in the spike trimeric interface, resulting in effective neutralization of SARS-CoV-2. The researchers are buoyant about the apt application of these, either alone or in synergy with other SARS-CoV-2 neutralizing antibodies, especially the ACE2-competing neutralizing antibodies. They may also be employed as integrant for creating bispecific or multispecific antibodies (12). Previously, He et al. (54) had demonstrated an augmented efficacy of oligomeric nanobodies, relative to monomeric nanobodies against MERS coronavirus RBD. Investigating the potential of such oligomeric nanobodies in the case of SARS-CoV-2 would be attention-grabbing.

These studies spark obvious anticipations and hopes for the potential application of nanobodies against COVID-19. The attributes of small size (almost one-fourth of the size of human antibodies) and simple structure, ease and comparatively lower cost, low immunogenicity and ability to display high affinity have endowed them with a special niche in the realm of therapeutics and rapid point-of-care diagnostics. Nanobodies seem to be quite efficient in trapping and stabilizing conformation-switchable targets in specific conformations, facilitating greater insight into biomolecular mechanisms and interactions. This could be of immense relevance to mine information on SARS-CoV-2 pathogenesis. Most importantly, highly stable VHHs could be nebulized and exploited for the development of inhalable prophylactic formulations, thereby ensuring straight delivery to the lungs- the combat zone. Another merit lies in the plausibility of stockpiling the VHHs without trade-off in their stability even after extended storages and using them as therapeutic choices in case of disasters like COVID-19. To conclude, I do hope that the incessant and concerted research endeavors would surely pave the way to a safer world, liberated from the grasp of SARS-CoV-2 and akin.

## Author Contributions

RK reviewed the literature, critically analyzed it and authored the article.

## Conflict of Interest

The author declares that the research was conducted in the absence of any commercial or financial relationships that could be construed as a potential conflict of interest.
